# Pan-PPAR Agonist, Bezafibrate, Restores Angiogenesis in Hindlimb Ischemia in Normal and Diabetic Rats

**DOI:** 10.1155/2012/637212

**Published:** 2012-06-03

**Authors:** M. Khazaei, E. Salehi, B. Rashidi

**Affiliations:** ^1^Department of Physiology, Isfahan University of Medical Sciences, Hezar Jarib Ave, Isfahan, Iran; ^2^Department of Anatomy, Isfahan University of Medical Sciences, Isfahan, Iran

## Abstract

*Introduction*. The aim of this study was to investigate the effect of bezafibrate as a pan-PPAR agonist on angiogenesis and serum nitrite, the main metabolite of nitric oxide (NO), vascular endothelial growth factor (VEGF) and VEGF receptor-2 (VEGFR-2) concentrations in hindlimb ischemia model of normal and type I diabetic rats. *Methods*. 28 male Wistar rats were divided into control and diabetic groups. Then, all rats underwent unilateral hindlimb ischemia. After recovery, they were randomly assigned to one of the following experimental groups: (1) control; (2) control + bezafibrate (400 mg/kg/day); (3) diabetic; (4) diabetic + beztafibrate. After three weeks, blood samples were taken and capillary density was evaluated in the gasterocnemius muscle of ischemic limb. *Results*. Bezafibrate increased capillary density and capillary/fiber ratio in ischemic leg of diabetic and control rats (*P* < 0.05). Serum VEGF and VEGFR-2 concentrations did not alter after bezafibrate administration, however, serum nitrite concentration was significantly higher in bezafibrate-treated groups than non-treated groups (*P* < 0.05). *Discussion*. It seems that bezafibrate, as a pan PPAR agonist, restores angiogenesis in hindlimb ischemic diabetic animals and is useful for prevention and/or treatment of peripheral artery disease in diabetic subjects.

## 1. Introduction

Peroxisome proliferator-activated receptors (PPARs) are ligand-inducible transcription factors that regulate expression of genes involved in some biological effects including lipid metabolism, inflammatory responses, and glucose homeostasis [1]. There are three isotypes of PPAR superfamily: PPAR*α*, PPAR*β*, and PPAR*γ*. Today, PPAR agonists have clinical importance in management of dyslipidemia (such as clofibrate, a PPAR*α* agonist) and reducing insulin resistance and antidiabetic activity (such as rosiglitazone, a PPAR*γ* agonist). Bezafibrate, a PPAR*α* agonist, is high-affinity ligand of PPAR*γ* and PPAR*β* and is considered as a pan-PPAR agonist [2, 3]. Bezafibrate is more efficient in reducing body weight and blood glucose than fenofibrate in overweight mice fed with high-fat diet [4]. It also raises HDL, reduces triglyceride, and improves insulin sensitivity in diabetic subjects [5, 6].

Diabetes is associated with several cardiovascular abnormalities including abnormal angiogenesis [7]. Enhanced or insufficient angiogenesis plays a role in some complications of diabetes including diabetic retinopathy or impaired wound healing, respectively [8]. In recent years, it is suggested that PPARs may be involved in regulating angiogenesis [9]; however, the role of these receptors in angiogenesis process still remained unclear [9].

Angiogenesis is a growth of new blood vessels from preexisting vessels and stimulated by tissue hypoxia via activation of hypoxia-inducible factor [10]. Then, activation of transcription of several angiogenic factors occurs. Vascular endothelial growth factor (VEGF) is a 45 kD glycoprotein which is involved in physiological and pathological angiogenesis [11]. VEGF has two tyrosine kinase receptors: VEGF receptor-1 (VEGFR-1) and VEGF receptor-2 (VEGFR-2). VEGFR-2 is the predominant effector of proangiogenic signaling in angiogenesis process [12]. Nitric oxide (NO) is another angiogenic factor which plays a key role in angiogenesis [13] especially in postischemic revascularization [14]. Since peripheral artery disease is one of the most important complications of diabetes, this study aimed to investigate the effect of bezafibrate as a pan-PPAR agonist on angiogenesis in hindlimb ischemia model of normal and type I diabetic rats.

## 2. Materials and Methods

### 2.1. Animals

In total, 28 male Wistar rats (Pasteur Institute of Iran) at 8–10 weeks old were studied. The animals were kept two per cage in animal room with 12 h light/dark cycle and room temperature between 20 and 25°C and had free access to water and food *ad libitum*. The ethical committee of the authors' institution approved all experimental protocol.

### 2.2. Hindlimb Ischemia Model and Treatment Groups

Diabetes was induced by single dose of intraperitoneal injection of streptozotocin (55 mg/kg). Control groups received normal saline injection with the same volume. After 48 h, blood glucose levels were measured and the animals with blood glucose level higher than 16.7 mmol/lit were considered as diabetic [15]. Then, all rats were anaesthetized by intraperitoneal injection of ketamine (75 mg/kg) and xylazine (7.5 mg/kg) and underwent unilateral hindlimb ischemia as previously described [16, 17]. For this purpose, femoral artery and all major branches were closed and excised. Then, the skin was closed with 3–0 surgical suture. Diabetic and control groups were randomly divided into two groups: treated with bezafibrate and nontreated groups. Bezafibrate was dissolved in corn oil and administered 400 mg/kg/day by gavage on day after operation [18]. Nontreated groups received the vehicle. The rats were assigned to one of the following experimental groups: (1) control; (2) control + bezafibrate; (3) diabetic; (4) diabetic + bezafibrate (*n* = 7 each). Before experiment (after randomization) and after three weeks of treatment, blood samples were taken and centrifuged at 3000 c/s for 20 minutes. Serum samples were poured in Eppendorf tubes and saved at −70°C for further analysis of serum nitrite, VEGF, and VEGFR-2 concentrations.

### 2.3. Serum Nitrite, VEGF, and VEGFR-2 Measurements

The serum concentration of nitrite, the main metabolite of NO, was assayed by griess reagent method (Promega Corp, USA) according to the manufacture's instruction. The serum levels of VEGF and VEGR-2 were measured using a sandwich enzyme immunoassay kit (R&D systems, USA). Serum lipids and blood glucose concentrations were measured using commercially available kits.

### 2.4. Measurement of Capillary Density

The animals were sacrified by cervical dislocation at 21 days after treatment, and capillary density was evaluated in the gasterocnemius muscle of ischemic limb. The samples were embedded in paraffin. Sections with 5 *μ*m thickness were stained with rat monoclonal antibody against murine CD31 (Abcam, Cambridge, UK). Capillary endothelial cells were identified by CD31-positive cells and counted by a light microscope. Ten microscopic fields (×400) from three different sections in each tissue block were randomly selected, and the number of capillaries was counted by two blinded observers. Capillary density was expressed as the number of capillaries per square millimeter. Capillary/muscle fiber ratio was also expressed, because muscle atrophy or interstitial edema may overestimate or underestimate capillary density.

### 2.5. Statistical Analysis

All data are expressed as mean ± SE. The Kolmogorov-Smirnov test for evaluation of normal distribution of data. One-way ANOVA using Tukey's post hoc test was used to compare data between groups. Bivariate correlations were calculated using Pearson's correlation coefficient. Paired data was analyzed by paired *t*-test. *P* value less than 0.05 was considered statistically significant.

## 3. Results

### 3.1. Serum Lipid Profile

All of the animals were survived after induction of hindlimb ischemia. Serum lipid profile of experimental groups is shown in Table 1. Our results showed that bezafibrate did not change the plasma levels of triglyceride (TG), high-density lipoprotein cholesterol (HDL-C), low-density lipoprotein cholesterol (LDL-C), and total cholesterol (TC) in control and diabetic animals (*P* > 0.05).

### 3.2. Serum Nitrite Concentration

**Figure 1:**
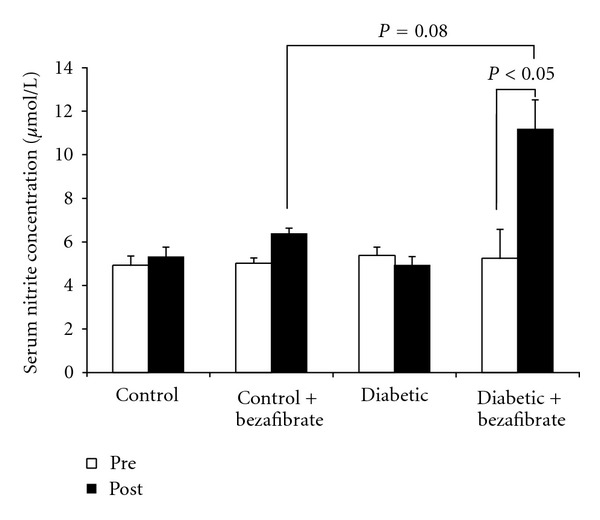
Serum nitrite concentration (*μ*mol/lit) before (pre) and after (post) experiment in the study groups. *n* = 7 each group.

Figure 1 illustrates serum nitrite concentration in all experimental groups. Results showed that diabetic animals had lower serum nitrite concentration than control group (*P* = 0.08). Also, the diabetic animals who received bezafibrate had higher serum nitrite concentration than non-treated group (*P* < 0.05).

### 3.3. Serum VEGF and VEGFR-2 Concentrations

**Figure 2:**
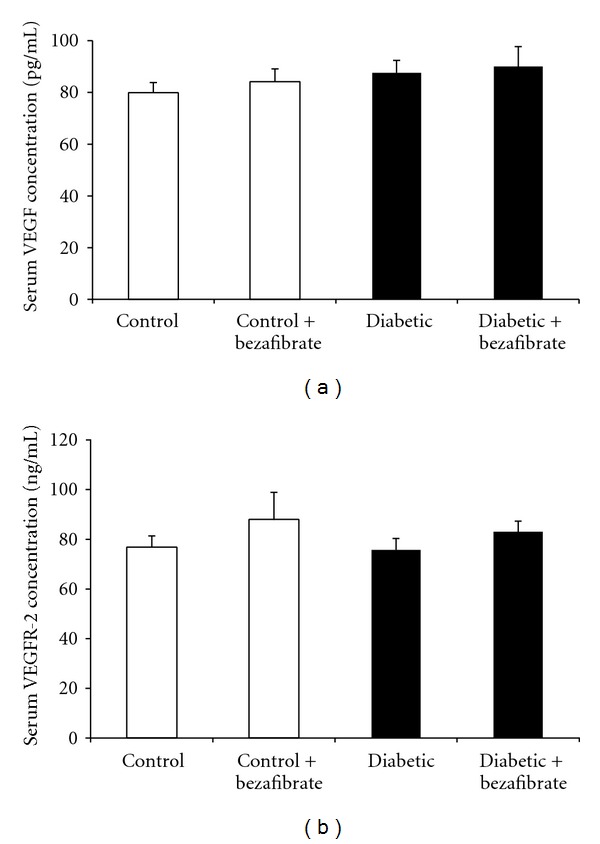
Serum VEGF (a) and VEGFR-2 (b) concentrations in experimental groups. *n* = 7 each group.

Results showed that there were no significant differences in serum VEGF and VEGFR-2 concentrations between diabetic and control groups (*P* > 0.05). Bezafibrate administration did not significantly change serum VEGF and VEGFR-2 values in control and diabetic animals (*P* > 0.05) (Figure 2).

### 3.4. Capillary Density

**Figure 3:**
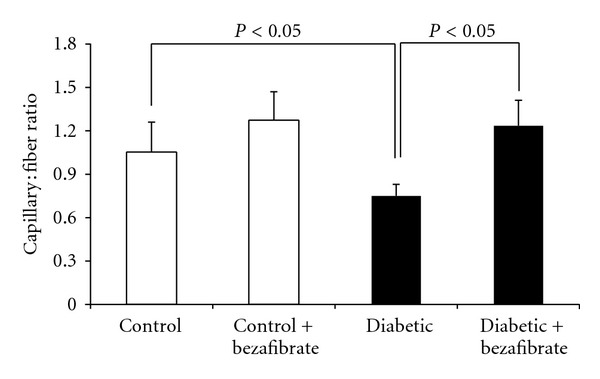
Effect of pan-PPAR agonist, bezafibrate, on capillary density/fiber ratio of hindlimb ischemia. *n* = 7 each group.

**Figure 4:**
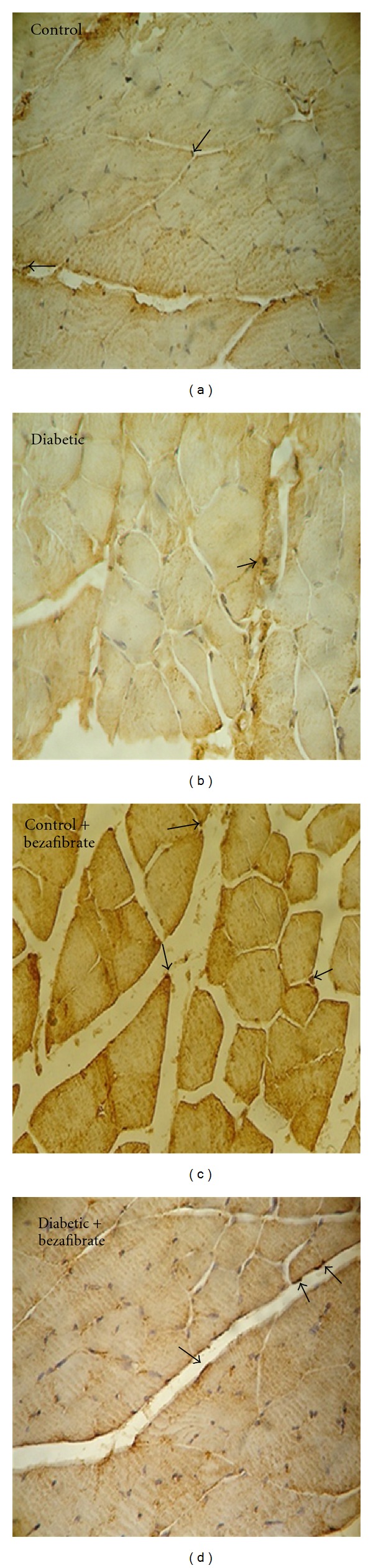
Representative photographs of immunohistochemical staining (×400) with anti-CD31 monoclonal antibody in experimental groups. Arrows indicates CD31-positive cells.

Figure 3 illustrates that the capillary density/muscle fiber ratio in ischemic gastrocnemius muscle of diabetic animals was significantly lower than control (*P* < 0.05). Capillary/fiber ratio was also significantly lower in diabetic than control group (*P* < 0.05). Bezafibrate significantly restored capillary density and capillary/fiber ratio in both diabetic and control groups (*P* < 0.05). Samples of immunohistochemical stained with anti-CD31 monoclonal antibody are shown in Figure 4.

### 3.5. Correlation Analysis

In the correlation analysis, we found that capillary density in skeletal muscle tissue was positively correlated with the serum nitrite level (*r* = 0.55).

## 4. Discussion

Diabetes is one of the most important risk factors for development of peripheral artery disease [19], and therapeutic angiogenesis has been considered to improve tissue perfusion in these subjects. In this study, we found that neovascularization in hindlimb ischemic diabetic animals was lower than control and bezafibrate significantly restored capillary density and serum nitrite level in diabetic hindlimb ischemic rats.

Among many genes induced after hypoxia to increase new blood vessel growth, the VEGF family genes and NO synthase are most important [9]. VEGF is one the most important growth factors which affects all aspects of angiogenesis including matrix degradation and endothelial cell migration and proliferation [8, 11, 20]. VEGFR-2 is expressed primarily on endothelial cells and is the predominant effector of angiogenesis signaling [12]. The present study showed that bezafibrate administration did not change serum VEGF and VEGFR-2 concentrations, however, significantly increased serum nitrite, the main metabolite of NO, level. NO has a key role in physiological and pathological neovascularization process [13, 21]. NO regulates endothelial cell proliferation, migration, and apoptosis [22, 23]. It also mediates the angiogenic response to VEGF or other angiogenic factors [24].

In the recent years there has been increasing evidences that PPARs are involved in regulation of angiogenesis; however, the exact role of these receptors still remained controversial [9, 25, 26]. Bezafibrate is a high-affinity ligand for all three isoforms of PPARs and considered as a pan-PPAR agonist [2, 3]. In clinical studies, antidiabetic and antihyperlipidemic effects of bezafibrate have been documented [5, 6]. It also prevents ischemic heart injury and reduces coronary artery disease [27, 28]. In the present study, we found that bezafibrate restored angiogenesis in hindlimb ischemic diabetic rats. According to our knowledge, this is the first study in this model of angiogenesis in diabetic animals. Balance between pro- and antiangiogenic factors regulates angiogenesis process. PPARs may modulate angiogenesis through the action of some growth factors and cytokines [9]. Biscetti et al. suggested that the effect of PPARs on angiogenic process is dependent on VEGF activity [29]. In the present study, we found that bezafibrate did not significantly alter serum VEGF and VEGFR-2 concentrations in diabetic animals. Instead, it increased serum NO level in diabetic group. A clinical study in patients with metabolic syndrome demonstrated that bezafibrate reduced incidence of myocardial infarction [3]. The importance of NO in angiogenesis in hindlimb ischemia model has been documented in previous studies [14, 17]. Thus, with considering the positive correlation between serum nitrite level and capillary density, our results suggest the possible involvement of NO pathway in increased neovascularization after bezafibrate administration.

In conclusion, while bezafibrate, as pan-PPAR agonist, restores angiogenesis in hindlimb ischemic diabetic animals and may be beneficial for prevention or treatment of peripheral artery disease in diabetic subjects, further studies for evaluation of its impact on other angiogenic, antiangiogenic, and endothelial markers, especially in long-term use in human, are required.

## Figures and Tables

**Table 1:** Serum lipid profile before and after experiments. There were no significant differences between bezafibrate-treated and nontreated groups (*P* > 0.05).

Groups	Total cholesterol (mg/dL)		Triglyceride (mg/dL)		HDL-C (mg/dL)		LDL-C (mg/dL)	
	Before	After	Before	After	Before	After	Before	After

Control	66.00 ± 6.79	84.40 ± 8.74	93.16 ± 6.72	73.40 ± 5.88	28.33 ± 4.19	44.66 ± 5.01	25.56 ± 1.94	28.52 ± 3.66
Control + bezafibrate	84.40 ± 8.74	79.80 ± 9.35	63.40 ± 5.88	87.00 ± 6.77	44.66 ± 5.01	33.60 ± 4.24	19.63 ± 2.44	20.76 ± 2.56
Diabetic	70.33 ± 5.44	72.25 ± 7.06	81.80 ± 15.02	71.25 ± 12.37	26.00 ± 3.55	33.40 ± 2.76	19.63 ± 2.44	20.76 ± 2.56
Diabetic + bezafibrate	72.25 ± 7.06	85.42 ± 9.18	71.25 ± 12.37	93.75 ± 10.43	33.40 ± 2.76	37.14 ± 4.74	21.16 ± 2.78	24.20 ± 5.67

Data are expressed as mean ± SE.
